# Clinical significance of the modified Naples prognostic score in patients with stage II-III colon cancer undergoing curative resection: a retrospective study from the real world

**DOI:** 10.3389/fonc.2024.1403666

**Published:** 2024-09-16

**Authors:** Xiaopeng Li, Chen Cheng, Xiongwei Huo, Chenye Zhao, Hang Yuan, Gang Chen, Junhui Yu, Mingchao Mu, Xuejun Sun

**Affiliations:** ^1^ Department of General Surgery, the First Affiliated Hospital of Xi’an Jiaotong University, Xi’an, China; ^2^ Department of Gynecologic Oncology, the Shaanxi Provincial Cancer Hospital, Xi’an, China

**Keywords:** colon cancer, modified Naples prognostic score, prognostic factors, curative resection, nomogram

## Abstract

**Background:**

The Naples prognostic score (NPS) determined by the nutritional and inflammatory condition of an individual is attracting growing attention for predicting postoperative outcomes in a variety of malignancies. The study aimed to assess the clinical significance of a modified NPS (M-NPS) and establish and validate nomograms incorporating M-NPS in curative stage II-III colon cancer patients.

**Methods:**

We retrospectively analyzed 328 stage II-III colon cancer patients receiving radical surgical resection at our hospital from January 2011 to December 2016. Kaplan–Meier (KM) survival analysis and Cox regression analysis were executed for overall survival (OS) and cancer-specific survival (CSS). Independent predictive indicators were applied to develop nomograms. The model’s performance was evaluated using many different methods.

**Results:**

Of a total of 328 cases, 153 cases were in group 0, 145 in group 1, and 30 in group 2. In terms of OS or CSS, there were obvious differences between groups 0 and 1, and between groups 0 and 2. Age, obstruction, N stage, gross tumor type, and M-NPS group were independent prognostic indicators for OS, while obstruction, gross tumor type, M-NPS group, and N stage were independent predictive parameters for CSS. Furthermore, the training and validation sets were randomly allocated among a cohort of 328 patients. OS and CSS prediction nomograms were developed. In the training and validation cohort, the C-index and ROC analysis showed good discrimination, calibration curves exhibited an excellent level of consistency between model-predicted survival and actual survival outcomes, and DCA curves demonstrated good clinical performance.

**Conclusion:**

M-NPS is a reliable survival predictor in patients with curative stage II-III colon cancer. Nomograms incorporating M-NPS for OS and CSS have good predictive performance and clinical utility.

## Introduction

Colorectal cancer (CRC) ranks third in prevalence and is the second leading cause of cancer deaths globally, according to recent statistics (1). Smoking, alcohol, obesity, diabetes, poor diets and low physical activity increase CRC risk (2, 3). Approximately 50% of colon cancer patients are stage II or III at the time of diagnosis (4). Radical surgery is the primary therapeutic approach for II-III colon cancer (4, 5). Although surgical resection significantly improves the survival of stage II-III patients, postoperative prognostic monitoring of these individuals continues to be a great challenge as a result of the disease’s highly heterogeneous nature. The current widely accepted prognostic assessment of colon cancer is based on postoperative pathological indicators (6). These histological factors can be obtained for assessment only after surgery. Therefore, the identification of a preoperatively available prognostic marker is extremely important for physicians’ clinical decision-making and prognostic risk stratification.

It is generally accepted that the prognosis of cancer individuals is influenced not only by the biology of the cancer but also by host-related conditions (7, 8). Inflammation, a leading hallmark of cancer, has a significant impact on tumorigenesis and progression (9). The cytokine release mediated by the NLRP3 inflammasome in various cell types contributes to the formation of an inflammatory tumor microenvironment through autocrine and paracrine signaling mechanisms (10). In colon cancer, activation of NLRP3 functions as a tumor suppressor by mediating the production of IL-18, which reinforces the killing activity of natural killer cells against metastatic tumor cells or down-regulates IL-22BP to suppress tumorigenesis (10, 11). The nutritional, inflammatory, and immune conditions of individuals are thought to be tightly associated with the outcome of CRC (12–18). Prior research has indicated that a variety of serum inflammation-related biomarkers can be beneficial for assessing the outcome in CRC (17), such as neutrophil-to-lymphocyte ratio (NLR) (19, 20), platelet-to-lymphocyte ratio (PLR) (21, 22) and lymphocyte-to-monocyte ratio (LMR) (23, 24). Additionally, several scoring systems, including the controlling nutritional status score (25, 26), the systemic inflammation score (27), the prognostic nutritional index (12, 28), and the Glasgow prognostic score (27, 29), had been confirmed as promising predictive markers for CRC outcome. NPS, a new scoring system to assess cancer outcomes, has attracted widespread attention. NPS based on inflammatory and nutritional status, first defined by Gennaro et al., is constructed from total cholesterol (CHOL), serum albumin (ALB), LMR, and NLR (30). Currently, the prognostic significance of NPS in CRC, obstructive CRC, metastatic CRC (mCRC) and rectal cancer has been reported by related studies (28, 30–32). The clinical significance of NPS in stage II-III patients, particularly in those receiving radical resection, has yet to be documented.

This study aims to construct a M-NPS for stage II-III patients, assess its prognostic value, and create a nomogram to forecast survival outcomes.

## Patients and methods

### Patient cohort

**Figure 1:**
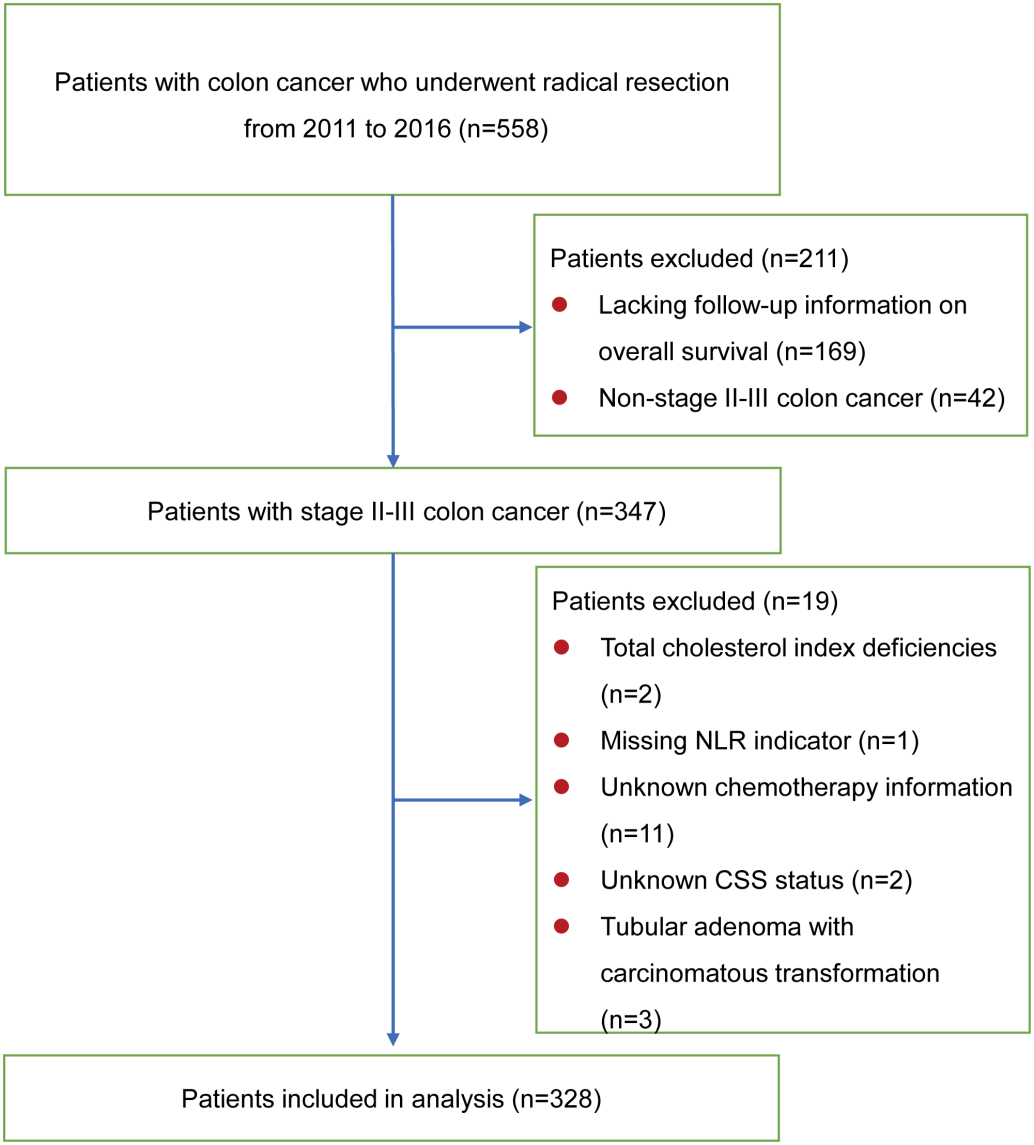
Flowchart for identifying the study population. CSS, cancer-specific survival.

This study ultimately enrolled 328 stage II-III colon cancer patients, who underwent curative surgical resection at the Department of General Surgery, the First Affiliated Hospital of Xi’an Jiaotong University from January 2011 to December 2016 (**Figure 1**). All subjects were diagnosed with preoperative biopsy or postoperative pathological examination. All patients were diagnosed with colon cancer and not in combination with other cancers, and none of them underwent neoadjuvant chemotherapy before surgery. The following were the exclusion criteria: definite inflammatory or hematologic diseases affecting immune or nutritional status; unknown prognostic information due to lost to follow-up; missing blood test results within one week before surgery. Patients were followed up via outpatient services or telephone interviews to obtain their health condition. The follow-up concluded in December 2022. The outcome was specified as either OS or CSS. OS was described as the interval between the surgical procedure and mortality resulting from any cause. The period from surgery to mortality as a result of recurrence or metastasis of the primary malignancy was referred to as CSS. For CSS, a patient’s death was defined as a non-endpoint event if the cause of death was not due to cancer. Cases lacking an outcome event were censored in the analysis.

### Data gathering

The clinicopathological indicators extracted from the patient’s electronic health data include age, sex, underlying disease, anemia, tumor location, American Society of Anesthesiologists (ASA) classification, surgical approach, ascites, intestinal perforation, obstruction, tumor size, gross tumor type, pathology type, differentiation, N stage, T stage, TNM stage, lymph node dissection, perineural invasion, vessel invasion and chemotherapy. Laboratory examinations within one week before surgery include ALB, CHOL, total counts of neutrophils, lymphocytes, and monocytes.

### Construction of M-NPS

**Figure 2:**
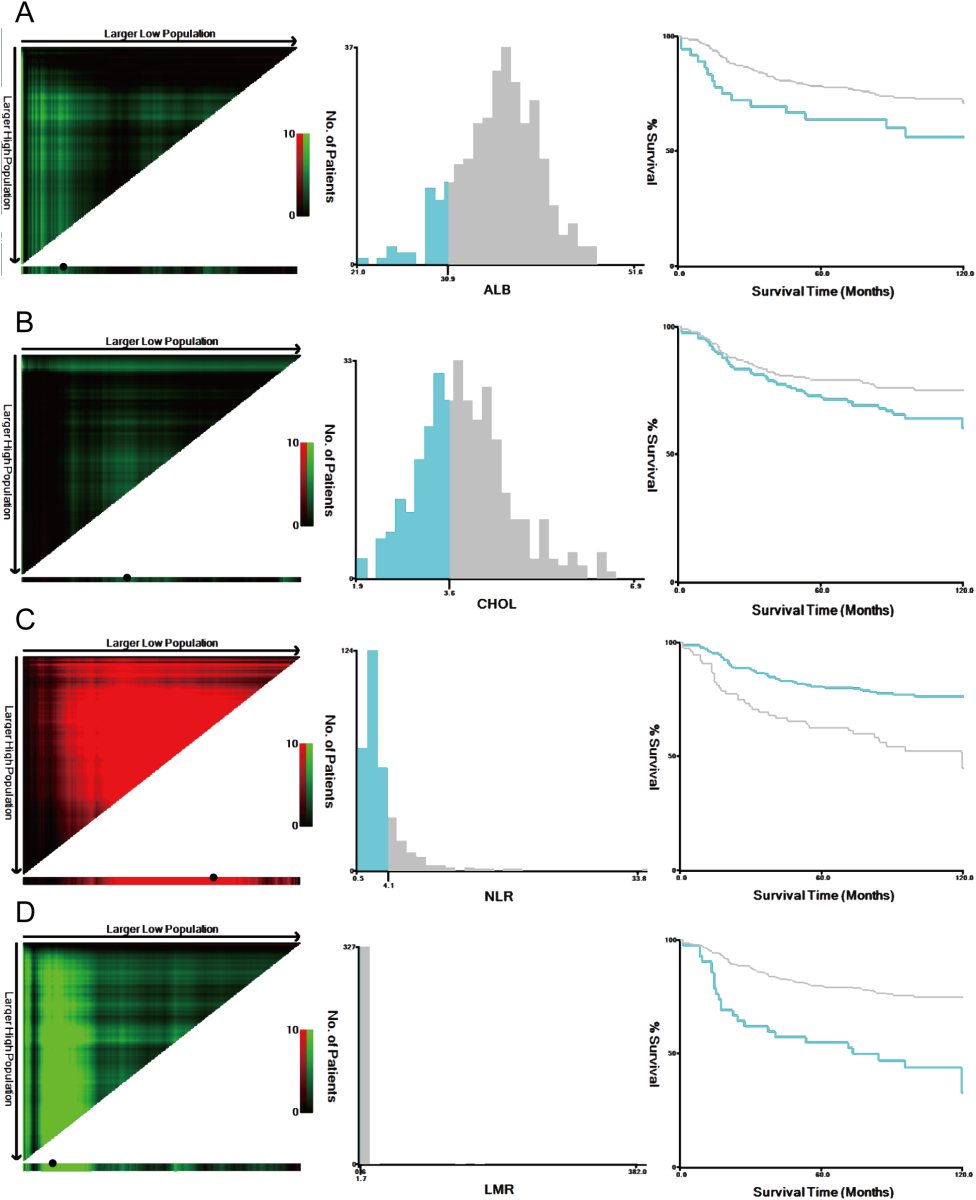
Optimal cutoff values were identified by the X-Tile analysis. The plots showed the χ 2 log‐rank values produced when dividing the cohort into two groups with optimal cut-points. Red coloration of cut points indicates an inverse correlation with survival, while green ones positively correlate. The optimal cutoff values highlighted by the black circles in the left panels are displayed in histograms of the entire cohort (middle panels), and Kaplan–Meier plots are shown in the right panels. For overall survival, the optimal thresholds for ALB **(A)**, CHOL **(B)**, NLR **(C)**, and LMR **(D)** were identified as 30.9 g/L, 3.6 mmol/L, 4.06, and 1.72, respectively.

M-NPS is a scoring system calculated from four factors: ALB, CHOL, LMR and NLR. Given that traditional NPS failed to exhibit substantial clinical utility in the study population, X-Tile 3.6.1 software (Yale University, New Haven, USA) (33) was employed to identify the most suitable threshold value for the four indicators according to the maximum chi-square and the lowest *P* values of log-rank tests (**Figure 2**). **Table 1** provides the comprehensive scoring criteria for the M-NPS. Patients were classified into three groups according to their total M-NPS scores: group 1 for scores of 0, group 2 for scores of 1-2, and group 3 for scores of 3-4.

**Table 1:** Scoring criteria of the modified Naples prognostic score (M-NPS).

Variables	Cutoff value	Points
Serum albumin (g/L)	>30.9	0
	≤30.9	1
Total cholesterol (mmol/L)	>3.6	0
	≤3.6	1
NLR	>4.06	1
	≤4.06	0
LMR	>1.72	0
	≤1.72	1

NLR, neutrophil-to-lymphocyte ratio; LMR, lymphocyte-to-monocyte ratio.

### Procedures of treatment

All curative surgeries for patients enrolled in the study were conducted by a skilled surgical team. They underwent open resection or laparoscopic operation, subject to the location of the disease and the surgical team’s preference. Adjuvant chemotherapy was implemented within two months postoperatively according to pathologic TNM staging results and the patient’s condition. Some patients discontinue chemotherapy midway because they cannot tolerate the side effects.

### Statistical analysis

The medians and interquartile ranges (IQRs) of continuous variables are shown, while categorical variables are described as percentages and numbers. Continuous variables were compared by the *Kruskal*-Waills test or the *Mann-Whitney U* test. For comparison of categorical variables between two or more groups, *chi-square* or *Fisher’s* exact tests were implemented. The survival and survminer R packages were employed to plot KM survival curves and compared their survival rates with a *log-rank* test. Independent prognostic analysis for OS or CSS was determined by multivariate Cox regression. A multivariate analysis was performed only on parameters with P<0.05 in a univariate analysis. The dataset was randomly divided in 6.5:3.5 into training and validation sets using the sample function. Independent prognostic variables were employed to develop nomograms in the training set using the rms R package. The model discrimination can be assessed with the C-index and the ROC curve. The calibration curve was generated with the rms R package to evaluate the prediction ability of the model. Additionally, the DCA was employed to evaluate the clinical efficacy of the model using the ggDCA R package. Version 4.3.2 of the R software was used to conduct all statistical analyses. Statistical tests with P< 0.05 are significantly difference.

## Results

### Patients’ baseline features and connection between M-NPS and clinicopathological parameters of patients

**Figure 1** displays the inclusion of 328 patients in the ultimate statistical analysis. Of the 328 patients, the age at diagnosis had a median value of 62 years. The research comprised 165 male and 163 female participants. The study subjects consisted of 113 patients with underlying diseases and 215 patients without underlying diseases. There were 148 patients with left hemicolon cancers, while 180 patients had right hemicolon cancers. 199 patients received open resection and 129 underwent laparoscopic surgery (**Table 2**). The median OS and CSS were both 86 months. Based on M-NPS scores, the cohort was stratified into three groups: 153 patients in group 0, 145 in group 1, and 30 in group 2. Of all clinicopathologic features, male, no underlying disease, open surgery and larger tumor size were associated with higher M-NPS scores. Lower ASA classification, ascites, no intestinal perforation, no obstruction and chemotherapy were related to lower M-NPS scores (**Table 2**). In addition, ALB, CHOL and LMR were all negatively correlated with M-NPS scores, whereas NLR was positively related to M-NPS scores (**Table 2**).

**Table 2:** Association of modified Naples prognostic score and clinicopathological characteristics in 328 stage II-III colon cancer patients.

Variables	Group 0 (*n* = 153)	Group 1 (*n* = 145)	Group 2 (*n* = 30)	*P*-value
Age, M (Q_1_, Q_3_)	62 (52,71)	63 (49,71)	65 (56,75)	0.302
Sex, *n* (%) Female Male	98 (64.1) 55 (35.9)	53 (35.9) 93 (64.1)	13 (43.3) 17 (56.7)	<0.001
Underlying disease, *n* (%) No Yes	91 (59.5) 62 (40.5)	106 (73.1) 39 (26.9)	18 (60.0) 12 (40.0)	0.037
Anemia, *n* (%) No Yes	69 (45.1) 84 (54.9)	54 (37.2) 91 (62.8)	11 (36.7) 19 (63.3)	0.343
Tumor location, *n* (%) Left hemicolon Right hemicolon	79 (51.6) 74 (48.4)	55 (37.9) 90 (62.1)	14 (46.7) 16 (53.3)	0.059
ASA, *n* (%) 1/2 3/4	119 (77.8) 34 (22.2)	100 (69.6) 45 (30.4)	15 (50.0) 15 (50.0)	0.006
Surgical approach, *n* (%) Laparoscopic Open	71 (46.4) 82 (53.6)	50 (34.5) 95 (65.5)	8 (26.7) 22 (73.3)	0.036
Ascites, *n* (%) No Yes	138 (90.2) 15 (9.8)	120 (82.8) 25 (17.2)	18 (60.0) 12 (40.0)	<0.001
Intestinal perforation, *n* (%) No Yes	153 (100.0) 0 (0.0)	141 (97.2) 4 (2.8)	27 (90.0) 3 (10.0)	0.003
Obstruction, *n* (%) No Yes	115 (75.2) 38 (24.8)	94 (64.8) 51 (35.2)	12 (40.0) 18 (60.0)	<0.001
Tumor size, n (%) ≤ 5 cm > 5 cm	108 (70.6) 45 (29.4)	82 (56.5) 63 (43.5)	10 (33.3) 20 (66.7)	<0.001
Gross tumor type, *n* (%) Infiltrative Ulcerated Elevated	5 (3.3) 107 (69.9) 41 (26.8)	7 (4.8) 99 (68.3) 39 (26.9)	2 (6.7) 14 (46.6) 14 (46.7)	0.153
Pathology type, *n* (%) Adenocarcinoma Mucinous adenocarcinoma Mixed	132 (86.3) 9 (5.9) 12 (7.8)	115 (79.3) 18 (12.4) 12 (8.3)	24 (80.0) 3 (10.0) 3 (10.0)	0.291
Differentiation, *n* (%) Well Moderately Poorly	3 (2.0) 135 (88.2) 15 (9.8)	5 (3.5) 125 (86.2) 15 (10.3)	0 (0.0) 23 (76.7) 7 (23.3)	0.248
T stage, *n* (%) T2/ T3 T4	23 (15.0) 130 (85.0)	20 (13.8) 125 (86.2)	4 (13.3) 26 (86.7)	0.942
N stage, *n* (%) N0 N1 N2	100 (65.4) 44 (28.8) 9 (5.8)	86 (59.3) 41 (28.3) 18 (12.4)	21 (70.0) 6 (20.0) 3 (10.0)	0.298
Lymph node dissection, *n* (%) < 12 ≥ 12	40 (26.1) 113 (73.9)	26 (17.9) 119(82.1)	10 (33.3) 20 (66.7)	0.094
Perineural invasion, *n* (%) No Yes	146 (95.4) 7 (4.6)	136 (93.8) 9 (6.2)	28 (93.3) 2 (6.7)	0.790
Vessel invasion, *n* (%) No Yes	144 (94.1) 9 (5.9)	138 (95.2) 7 (4.8)	29 (96.7) 1 (3.3)	0.819
Chemotherapy, *n* (%) No Yes	47 (30.8) 106 (69.3)	57 (39.3) 88 (60.7)	18 (60.0) 12 (40.0)	0.008
ALB (g/L), M (Q_1_, Q_3_)	37.6 (35.5, 40.5)	36.3 (32.9, 38.9)	30.2 (28.7, 33.5)	<0.001
CHOL (mmol/L), M (Q_1_, Q_3_)	4.3 (3.9, 4.7)	3.4 (3.1, 3.8)	3.1 (2.9, 3.3)	<0.001
NLR, M (Q_1_, Q_3_)	2.0 (1.5, 2.6)	2.8 (2.0, 4.4)	6.1 (5.0, 7.6)	<0.001
LMR, M (Q_1_, Q_3_)	4.4 (3.2, 5.8)	3.0 (2.1, 4.5)	1.6 (1.2, 1.8)	<0.001

M, Median; Q_1_, 1st Quartile; Q_3_, 3st Quartile.

### Patient’s prognosis (OS and CSS) according to M-NPS

**Figure 3:**
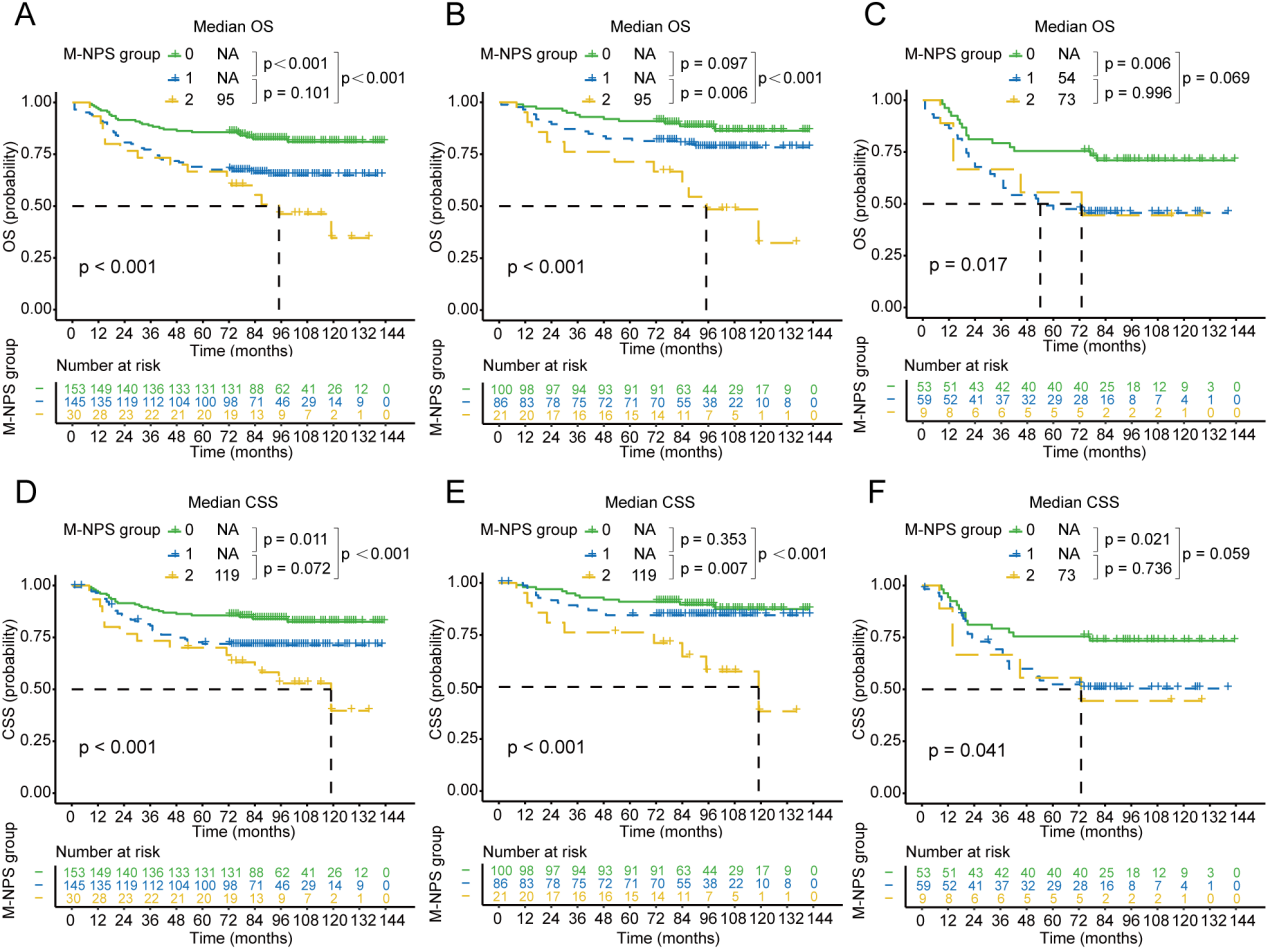
Kaplan–Meier survival curves of OS and CSS for each M-NPS group. Differences in OS among the three groups in patients with stage II-III **(A)**, II **(B)**, and III **(C)** colon cancer. Differences in CSS among the three groups in patients with stage II-III **(D)**, II **(E)**, and III **(F)** colon cancer. OS, overall survival; CSS, cancer-specific survival.

Survival analysis of OS and CSS based on M-NPS grouping was performed and survival curves were plotted in **Figure 3**. For OS in stage II-III patients, the median survival for group 2 was 95 months. Group 0 had more favorable survival than both group 1 and group 2 (**Figure 3A**). Further stratified survival analysis using staging showed that in comparison to group 0 and group 1, group 2 had the poorest prognosis for stage II patients (**Figure 3B**). In stage III patients, group 0 had superior survival than group 1 (**Figure 3C**), and a significant survival difference was not observed between group 1 and 2, nor between group 0 and 2. In terms of CSS for stage II-III patients, the median survival time in group 2 was 119 months. Group 0 had the best survival compared to group 1 and group 2 (**Figure 3D**). Similarly, among stage II patients, group 2 had poorer CSS compared to those in group 1 and 0 (**Figure 3E**). For stage III patients, group 0 had the most favorable CSS, and the remaining two groups did not appear to differ markedly in survival (**Figure 3F**).

### Univariate and multivariate analyses of prognostic factors for OS and CSS

We conducted univariate and multivariate Cox regression analysis on OS for stage II-III patients. The results are displayed in **Table 3**. OS had a strong association with the M-NPS group, as indicated by the univariate analysis. Additional multivariate analysis revealed that the M-NPS group was an independent risk indicator for OS. Group 1 and group 2 had worse OS compared to group 0 [G1 vs G0: 1.73 (1.05–2.86); G2 vs G0: 2.55 (1.29–5.03)]. Other independent prognostic risk indicators included age, obstruction, gross tumor type, and N stage.

**Table 3:** Univariate and multivariate analyses of the prognostic factor for OS.

Variables	Univariate analysis		Multivariate analysis	
	HR (95% CI)	*P*-value	HR (95% CI)	*P*-value
Age	1.03 (1.02-1.05)	<0.001	1.03 (1.01-1.05)	0.013^*^
Sex Female Male	Reference 1.30 (0.86-1.97)	0.205		
Underlying disease No Yes	Reference 1.16 (0.76-1.76)	0.495		
Anemia No Yes	Reference 1.01 (0.67-1.53)	0.946		
Tumor location Right hemicolon Left hemicolon	Reference 0.95 (0.63-1.42)	0.788		
ASA 1/2 3/4	Reference 2.23 (1.48-3.36)	<0.001	Reference 1.21 (0.73-2.01)	0.453
Surgical approach Laparoscopic Open	Reference 1.58 (1.02-2.47)	0.042	Reference 1.33 (0.83-2.13)	0.230
Ascites No Yes	Reference 2.03 (1.27-3.26)	0.003	Reference 0.86 (0.49-1.50)	0.585
Intestinal perforation No Yes	Reference 1.98 (0.63-6.25)	0.246		
Obstruction No Yes	Reference 2.51 (1.67-3.77)	<0.001	Reference 2.21 (1.38-3.52)	0.001^*^
Tumor size ≤ 5 cm > 5 cm	Reference 1.00 (0.66-1.52)	0.997		
Gross tumor type Infiltrative Ulcerated Elevated	Reference 0.67 (0.29-1.54) 0.29 (0.11-0.75)	0.347 0.011	Reference 0.49 (0.20-1.20) 0.23 (0.08-0.61)	0.120 0.003^*^
Pathology type Adenocarcinoma Mucinous Mixed	Reference 0.88 (0.43-1.83) 0.83 (0.38-1.80)	0.738 0.641		
Differentiation Well Moderately Poorly	Reference 0.71 (0.22-2.24) 0.71 (0.19-2.57)	0.556 0.599		
T stage T2 & T3 T4	Reference 1.16 (0.63-2.13)	0.623		
N stage N0 N1 N2	Reference 2.07 (1.30-3.29) 5.18 (3.02-8.87)	0.002 <0.001	Reference 1.92 (1.16-3.16) 4.57 (2.56-8.15)	0.011^*^ <0.001^*^
Lymph node dissection < 12 ≥ 12	Reference 0.81 (0.51-1.28)	0.370		
Perineural invasion No Yes	Reference 2.09 (1.01-4.32)	0.047	Reference 1.85 (0.85-4.02)	0.120
Vessel invasion No Yes	Reference 2.51 (1.26-4.99)	0.009	Reference 2.03 (0.97-4.24)	0.059
Chemotherapy No Yes	Reference 0.52 (0.35-0.78)	0.002	Reference 0.77 (0.47-1.26)	0.300
M-NPS Group 0 1 2	Reference 2.21 (1.39-3.53) 3.56 (1.92-6.61)	<0.001 <0.001	Reference 1.73 (1.05-2.86) 2.55 (1.29-5.03)	0.031^*^ 0.007^*^

NPS, Naples prognostic score; M-NPS, modified NPS.

*Statistically significant.

In addition, the same analytical methods as for OS were executed for CSS (**Table 4**). CSS was notably correlated with the M-NPS group in the univariate analysis. CSS was independently influenced by the M-NPS group in multivariate analysis. Group 2 had worse CSS compared to group 0 [G2 vs G0: 2.52 (1.24–5.14)]. Obstruction, gross tumor type, and N stage were also independent prognostic risk parameters of CSS.

**Table 4:** Univariate and multivariate analyses of the prognostic factor for CSS.

Variables	Univariate analysis		Multivariate analysis	
	HR (95% CI)	*P*-value	HR (95% CI)	*P*-value
Age	1.03 (1.01-1.05)	0.002	1.02 (1.00-1.05)	0.052
Sex Female Male	Reference 1.37 (0.88-2.15)	0.166		
Underlying disease No Yes	Reference 1.05 (0.66-1.66,)	0.849		
Anemia No Yes	Reference 0.84 (0.54-1.31)	0.441		
Tumor location Right hemicolon Left hemicolon	Reference 1.05 (0.67-1.63)	0.843		
ASA 1/2 3/4	Reference 1.81 (1.15-2.84)	0.011	Reference 1.03 (0.59-1.81)	0.911
Surgical approach Laparoscopic Open	Reference 1.57 (0.97-2.53)	0.067		
Ascites No Yes	Reference 2.09 (1.26-3.48)	0.004	Reference 0.89 (0.48-1.63)	0.706
Intestinal perforation No Yes	Reference 2.43 (0.77-7.71)	0.132		
Obstruction No Yes	Reference 2.65 (1.70-4.12)	<0.001	Reference 2.38 (1.43-3.96)	0.001^*^
Tumor size ≤ 5 cm > 5 cm	Reference 1.03 (0.66-1.62)	0.887		
Gross tumor type Infiltrative Ulcerated Elevated	Reference 0.70 (0.28-1.74) 0.26 (0.09-0.74)	0.442 0.011	Reference 0.50 (0.19-1.35) 0.20 (0.06-0.59)	0.170 0.004^*^
Pathology type Adenocarcinoma Mucinous Mixed	Reference 0.93 (0.43-2.04) 0.99 (0.45-2.16)	0.864 0.984		
Differentiation Well Moderately Poorly	Reference 0.91 (0.22-3.71) 0.96 (0.21-4.46)	0.895 0.962		
T stage T2 & T3 T4	Reference 1.34 (0.67-2.69)	0.403		
N stage N0 N1 N2	Reference 2.15 (1.29-3.58) 6.06 (3.43-10.68)	0.003 <0.001	Reference 1.80 (1.05-3.10) 5.37 (2.91-9.91)	0.034^*^ <0.001^*^
Lymph node dissection < 12 ≥ 12	Reference 0.88 (0.53-1.47)	0.637		
Perineural invasion No Yes	Reference 2.47 (1.19-5.13)	0.016	Reference 2.05 (0.94-4.48)	0.071
Vessel invasion No Yes	Reference 2.29 (1.05-4.97)	0.037	Reference 1.98 (0.86-4.52))	0.107
Chemotherapy No Yes	Reference 0.57 (0.37-0.89)	0.014	Reference 0.73 (0.43-1.25)	0.249
M-NPS Group 0 1 2	Reference 1.91 (1.16-3.15) 3.35 (1.74-6.44)	0.011 <0.001	Reference 1.45 (0.85-2.49) 2.52 (1.24-5.14)	0.175 0.011^*^

NPS, Naples prognostic score; M-NPS, modified NPS.

*Statistically significant.

### Division of the dataset and construction of the nomogram

The total of 328 patients finally included were stochastically allocated into two cohorts: 213 in the training set and 115 in the validation set. All clinicopathological characteristics in two sets are listed in **Table 5**. No statistical difference in clinicopathological characteristics between the two cohorts proved that the allocation was completely randomized (**Table 5**).

**Table 5:** Patients’ clinicopathological characteristics in the training and validation cohort.

Variables	All patients (n = 328)	Training set (n = 213)	Validation set (n = 115)	*P*-value
Age, M (Q₁, Q₃)	62.00 (52, 72)	62.00 (52, 71)	62.00 (53, 74)	0.306
Sex, n (%)				0.790
Female	163 (49.70)	107 (50.23)	56 (48.70)	
Male	165 (50.30)	106 (49.77)	59 (51.30)	
Underlying disease, n (%)				0.378
No	215 (65.55)	136 (63.85)	79 (68.70)	
Yes	113 (34.45)	77 (36.15)	36 (31.30)	
Anemia, n (%)				0.241
No	134 (40.85)	92 (43.19)	42 (36.52)	
Yes	194 (59.15)	121 (56.81)	73 (63.48)	
Tumor location, n (%)				0.980
Right hemicolon	180 (54.88)	117 (54.93)	63 (54.78)	
Left hemicolon	148 (45.12)	96 (45.07)	52 (45.22)	
ASA, n (%)				0.790
1/2	234 (71.34)	153 (71.83)	81 (70.43)	
3/4	94 (28.66)	60 (28.17)	34 (29.57)	
Surgical approach, n (%)				0.855
Open	199 (60.67)	130 (61.03)	69 (60.00)	
Laparoscopic	129 (39.33)	83 (38.97)	46 (40.00)	
Ascites, n (%)				0.941
No	276 (84.15)	179 (84.04)	97 (84.35)	
Yes	52 (15.85)	34 (15.96)	18 (15.65)	
Intestinal perforation, n (%)				0.971
No	321 (97.87)	209 (98.12)	112 (97.39)	
Yes	7 (2.13)	4 (1.88)	3 (2.61)	
Obstruction, n (%)				0.390
No	221 (67.38)	147 (69.01)	74 (64.35)	
Yes	107 (32.62)	66 (30.99)	41 (35.65)	
Tumor size, n (%)				0.103
>5	128 (39.02)	90 (42.25)	38 (33.04)	
≤5	200 (60.98)	123 (57.75)	77 (66.96)	
Gross tumor type, n (%)				0.460
Elevated	94 (28.66)	63 (29.58)	31 (26.96)	
Infiltrative	14 (4.27)	7 (3.29)	7 (6.09)	
Ulcerated	220 (67.07)	143 (67.14)	77 (66.96)	
Pathology type, n (%)				0.958
Adenocarcinoma	271 (82.62)	176 (82.63)	95 (82.61)	
Mixed	27 (8.23)	17 (7.98)	10 (8.70)	
Mucinous adenocarcinomas	30 (9.15)	20 (9.39)	10 (8.70)	
Differentiation, n (%)				0.931
Moderately	283 (86.28)	183 (85.92)	100 (86.96)	
Poorly	37 (11.28)	25 (11.74)	12 (10.43)	
Well	8 (2.44)	5 (2.35)	3 (2.61)	
T stage, n (%)				0.245
T2/T3	47 (14.33)	27 (12.68)	20 (17.39)	
T4	281 (85.67)	186 (87.32)	95 (82.61)	
N stage, n (%)				0.128
N0	207 (63.11)	134 (62.91)	73 (63.48)	
N1	91 (27.74)	64 (30.05)	27 (23.48)	
N2	30 (9.15)	15 (7.04)	15 (13.04)	
Lymph Nodes, n (%)				0.519
<12	76 (23.17)	47 (22.07)	29 (25.22)	
≥12	252 (76.83)	166 (77.93)	86 (74.78)	
Perineural Invasion1, n (%)				0.240
No	310 (94.51)	199 (93.43)	111 (96.52)	
Yes	18 (5.49)	14 (6.57)	4 (3.48)	
Vessel invasion1, n (%)				0.587
No	311 (94.82)	203 (95.31)	108 (93.91)	
Yes	17 (5.18)	10 (4.69)	7 (6.09)	
Chemotherapy, n (%)				0.366
No	122 (37.20)	83 (38.97)	39 (33.91)	
Yes	206 (62.80)	130 (61.03)	76 (66.09)	
M-NPS group, n (%)				0.121
G0	153 (46.65)	101 (47.42)	52 (45.22)	
G1	145 (44.21)	88 (41.31)	57 (49.57)	
G2	30 (9.15)	24 (11.27)	6 (5.22)	

M, Median, Q₁, 1st Quartile, Q₃, 3st Quartile; M-NPS, modified Naples prognostic score.

**Figure 4:**
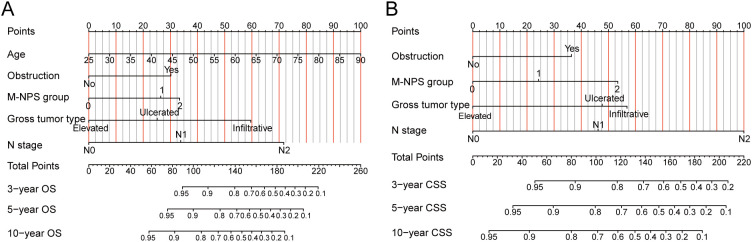
Nomograms for OS and CSS. **(A)** Nomogram for predicting 3-, 5- and 10-year OS. **(B)** Nomogram for predicting 3-, 5- and 10-year CSS. OS, overall survival; CSS, cancer-specific survival.

OS and CSS nomograms were created to forecast the survival rates at 3, 5, and 10 years, using independent parameters that influence OS and CSS, respectively (**Figure 4**). Nomograms can personalize the forecast of OS and CSS for each patient by calculating the sum of the corresponding score for each variable in the models.

### Evaluation of nomogram prediction models

The C-indexes for OS and CSS nomograms in the training cohort were 0.802 (0.747–0.857) and 0.783 (0.722–0.844), respectively. In the validation cohort, the C-indexes for OS and CSS nomograms were 0.742 (0.656–0.828) and 0.745 (0.649–0.841), respectively.

**Figure 5:**
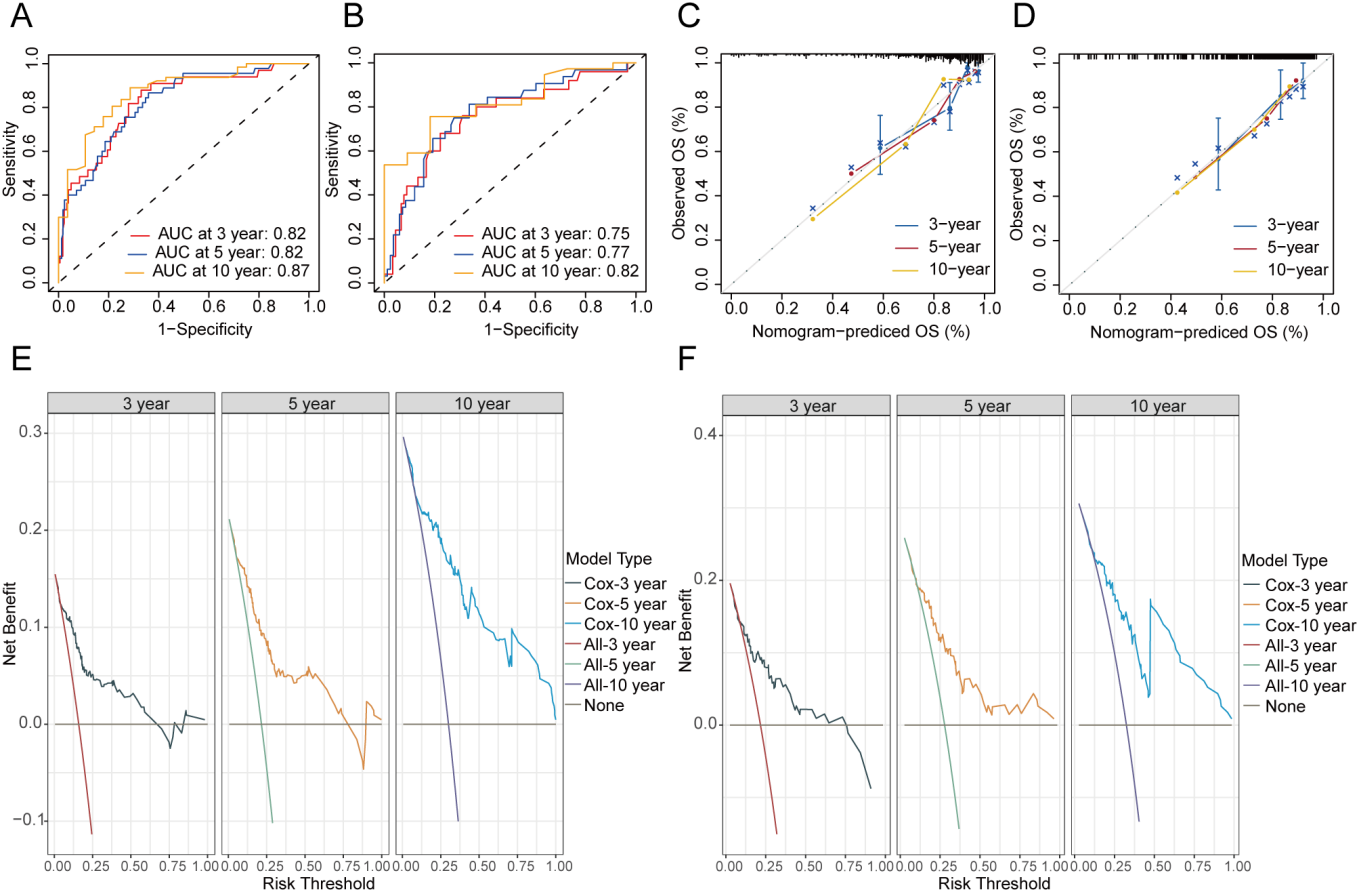
Evaluation of the nomogram of OS. ROC curves of nomograms for 3-, 5- and 10-year OS in the training **(A)** and validation cohort **(B)**. Calibration curves of the nomogram for the probability of 3-, 5- and 10-year OS in the training **(C)** and validation cohort **(D)**. DCA curves of the nomogram for 3-, 5- and 10-year OS in the training **(E)** and validation cohort **(F)**. OS, overall survival.

ROC analysis indicated that the nomogram for OS in the training cohort achieved AUC values of 0.82, 0.82, and 0.87 for the 3-, 5-, and 10-year predictions, respectively (**Figure 5A**). In the validation cohort, the predictive model for OS reached AUC values of 0.75, 0.77, and 0.82, respectively (**Figure 5B**). The nomogram’s calibration curves demonstrated that excellent alignment between predicted and actual 3-, 5-, and 10-year OS in both the training (**Figure 5C**) and validation sets (**Figure 5D**). In addition, DCA curves showed that the predictive model of OS had excellent clinical efficacy at 3, 5, and 10 years in clinical practice (**Figures 5E, F**).

**Figure 6:**
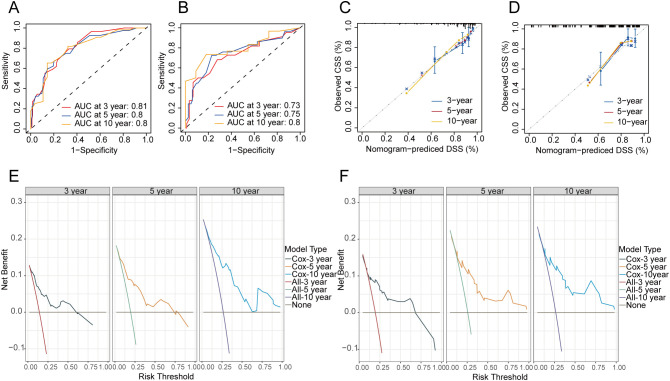
Evaluation of the nomogram of CSS. ROC curves of nomograms for 3-, 5- and 10-year CSS in the training **(A)** and validation cohort **(B)**. Calibration curves of the nomogram for the probability of 3-, 5- and 10-year CSS in the training **(C)** and validation cohort **(D)**. DCA curves for training **(E)** and validation cohort **(F)** at 3-, 5-, and 10-year CSS. CSS, cancer-specific survival.

At the same time, the CSS nomogram in the training set attained AUC values of 0.81, 0.80, and 0.80 for the 3-, 5-, and 10-year predictions, respectively (**Figure 6A**). In the validation set, the CSS nomogram got AUC values of 0.73, 0.75, and 0.80 for the 3-, 5-, and 10-year predictions, respectively (**Figure 6B**). In the training (**Figure 6C**) and validation cohort (**Figure 6D**), the nomogram’s calibration curves exhibited an excellent level of consistency between the model-forecasted CSS and the actual survival outcomes. Additionally, DCA curves for the nomogram of CSS indicated that the model had favorable clinical efficacy in both the training (**Figure 6E**) and validation cohort (**Figure 6F**).

## Discussion

Recently, research has been conducted extensively on the application of NPS to predict the outcome of numerous types of cancer due to the integration of multiple indicators of nutritional, inflammatory, and immune status (28, 30, 34–36). NPS thresholds in different cancer types and treatment contexts remain controversial. In our research, the X-Tile was employed to construct M-NPS based on stage II-III patients in the research center. Furthermore, we examined the connection between M-NPS and patients’ clinicopathological parameters and assessed the predictive significance of M-NPS for OS and CSS. Finally, OS and CSS nomograms containing M-NPS are established and validated.

NLR is a highly reliable indicator of systemic inflammation. The threshold value for NLR in NPS was originally defined by Gennaro et al. (30) as 4.44, and the cutoff value identified in this study was very close to 4.04. LMR is used as a marker of the host’s immunological response. We determined a cutoff value of 1.72 for LMR, however, the cutoff value for LMR in NPS is 2.96. In addition, the cutoff values for ALB and CHOL levels, which represent nutritional status, also differed from those defined by Gennaro et al. (30). The above differences may originate from the race, lifestyle, and dietary structure of Eastern and Western populations.

Accumulating evidence suggests that patients with CRC in a hyperinflammatory state portend an unfavorable prognosis, whereas patients in a good nutritional state show longer survival (15). Our study showed that colon cancer patients with low NLR, high ALB and high CHOL had a more favorable survival compared to those with high NLR, low ALB and low CHOL. Therefore, as recommended by the guidelines (37), an appropriate anti-inflammatory and nutritious diet is crucial in preventing the occurrence of colon cancer and reducing postoperative colon cancer deaths.

Our study revealed that obstruction was a significant predictive indicator for OS and CSS in stage II-III patients. Relevant studies have pointed out that obstruction as a marker of malignancy portends an unfavorable prognosis in CRC patients even after radical surgery (38–40). Obstruction causes dysbiosis and increased toxins, resulting in local and systemic inflammatory responses to remodel the tumor microenvironment (41, 42). Inflammation associated with cancer not only facilitates the growth of malignant tumors but can also reduce response to chemotherapeutic agents by inducing genetic instability (43). We have also found in the study that the gross tumor type was also an independent prognostic factor for the prognosis of patients with stage II-III colon cancer. As we all know, the gross tumor type reflects the biology of the cancer itself. Infiltrative colon cancers are the most malignant and elevated colon cancers are the least malignant. Previous studies have also revealed that gross tumor type could serve as a marker of survival and peritoneal metastasis in CRC (44, 45), which is consistent with our results.

NPS, as a novel and powerful scoring system, has been widely applied in the construction of prognostic nomograms for a variety of cancers, including gastric cancer (46), small cell lung cancer (47), esophageal squamous cell carcinoma (48, 49), cholangiocarcinoma (50), gallbladder cancer (51) and osteosarcoma (52). Currently, there are no reports related to the application of NPS to the prognostic nomogram of colon cancer. For the first time, we incorporated M-NPS into the predictive nomogram for colon cancer patients in stages II–III. Nomograms integrating M-NPS exhibited excellent predictive ability for 3, 5, and 10-year OS or CSS.

This study relies on a national clinical key specialty platform with a superb surgical team, sufficient surgical patients and advanced instrumentation. In addition, the professional follow-up process provides a strong warrant for the reliability of the study’s conclusions. However, there exist some constraints in our research. Initially, the retrospective study is susceptible to selection bias. Besides, the sample size of a single center is relatively limited and the number of outcome events is relatively few. Hence, the clinical significance of M-NPS has to be validated through prospective multicenter trials involving a substantial number of participants.

## Conclusions

Taken together, M-NPS is a robust survival predictor in curative stage II-III patients. Nomograms incorporating M-NPS for OS and CSS have good predictive performance and clinical utility.

## Data Availability

The data used in the study are not publicly available due to privacy restrictions but are available from the corresponding author on reasonable request.
